# Diffusion tensor and restriction spectrum imaging reflect different aspects of neurodegeneration in Parkinson’s disease

**DOI:** 10.1371/journal.pone.0217922

**Published:** 2019-05-31

**Authors:** Tuva R. Hope, Per Selnes, Irena Rektorová, Lubomira Anderkova, Nela Nemcova-Elfmarkova, Zuzana Balážová, Anders Dale, Atle Bjørnerud, Tormod Fladby

**Affiliations:** 1 Diagnostic Physics, Division of Radiology & Nuclear Medicine, Oslo University Hospital, Rikshospitalet, Oslo, Norway; 2 Department of Neurology, Akershus University Hospital, Loerenskog, Norway; 3 Institute of Clinical Medicine, Campus Ahus, University of Oslo, Oslo, Norway; 4 Central European Institute of Technology, CEITEC Masaryk University, Brno, Czech Republic; 5 First Department of Neurology, Medical Faculty, Masaryk University and St. Anne’s University Hospital, Brno, Czech Republic; 6 Department of Neurosciences, University of California, San Diego, La Jolla, California, United States of America; 7 Deparment of Radiology, University of California San Diego, San Diego, La Jolla, California, United States of America; 8 Deparment of Cognitive Sciences, University of California San Diego, San Diego, La Jolla, California, United States of America; 9 Department of Physics, University of Oslo, Oslo, Norway; University of North Carolina at Chapel Hill, UNITED STATES

## Abstract

To meet the need for Parkinson’s disease biomarkers and evidence for amount and distribution of pathological changes, MRI diffusion tensor imaging (DTI) has been explored in a number of previous studies. However, conflicting results warrant further investigations. As tissue microstructure, particularly of the grey matter, is heterogeneous, a more precise diffusion model may benefit tissue characterization. The purpose of this study was to analyze the diffusion-based imaging technique restriction spectrum imaging (RSI) and DTI, and their ability to detect microstructural changes within brain regions associated with motor function in Parkinson’s disease. Diffusion weighted (DW) MR images of a total of 100 individuals, (46 Parkinson’s disease patients and 54 healthy controls) were collected using b-values of 0–4000s/mm^2^. Output diffusion-based maps were estimated based on the RSI-model combining the full set of DW-images (Cellular Index (CI), Neurite Density (ND)) and DTI-model combining b = 0 and b = 1000 s/mm^2^ (fractional anisotropy (FA), Axial-, Mean- and Radial diffusivity (AD, MD, RD)). All parametric maps were analyzed in a voxel-wise group analysis, with focus on typical brain regions associated with Parkinson’s disease pathology. CI, ND and DTI diffusivity metrics (AD, MD, RD) demonstrated the ability to differentiate between groups, with strongest performance within the thalamus, prone to pathology in Parkinson’s disease. Our results indicate that RSI may improve the predictive power of diffusion-based MRI, and provide additional information when combined with the standard diffusivity measurements. In the absence of major atrophy, diffusion techniques may reveal microstructural pathology. Our results suggest that protocols for MRI diffusion imaging may be adapted to more sensitive detection of pathology at different sites of the central nervous system.

## Introduction

Parkinson’s disease (PD) is a slowly progressing neurodegenerative disorder, affecting both motor function and cognition. Accurate biomarkers for PD diagnosis and progression are scarce. PD is primarily diagnosed clinically after the pathology has reached an advanced stage, and confirmed post-mortem by loss of dopamine neurons in the substantia nigra and the presence of intracellular aggregates of α-synuclein called Lewy bodies [1]. Formation of Lewy bodies, loss of neurites, loss of dopamine-containing neurons and gliosis in substantia nigra is an early phenomenon in PD, whereas cortical pathology is thought to precede major subcortical affection on Dementia with Lewy Bodies [2, 3]. Lewy body pathology spreads from the brainstem and subcortical structures, to related areas with eventual allo- and neocortical involvement [4]. α-synuclein deposition is considered the core pathological substrate of PD. In addition to accumulation in Lewy-bodies, a major deposition site of central nervous system (CNS) oligomeric α-synuclein is in the presynapse, accompanied by transsynaptic dendritic spine loss [5, 6], suggesting widespread pathology. MRI studies have indicated that slight grey matter loss is involved in PD cognitive decline [7]. In PD with normal cognition, there is slight volume decrease in the frontoparietal regions and also increase in the midbrain and cerebellum. There is also a second pattern with medial temporal lobe atrophy more closely coupled to cognitive decline [8], and we recently described cortical metabolic changes associated with biomarker changes in temporo-occipital and frontal regions [9].

To meet the need for PD biomarkers and evidence for amount and distribution of pathological changes, MRI diffusion tensor imaging (DTI) techniques have been explored. By measuring diffusion of water molecules in the brain, this technique gives information on neuronal integrity which may be linked to disease state. A number of studies have reported that the DTI derived indices fractional anisotropy (FA) and mean diffusivity (MD) in the substantia nigra and other brain structures associated with PD pathology differentiate between PD and controls with decreased FA and increased MD in PD. However, there are conflicting reports. Some more recent studies have reported an increase in FA in early PD, while several other studies have not found any differences. For example; studies manually delineating regions of interest have found decreased FA in the substantia nigra compared to controls [10–16], or asymmetrical substantia nigra FA [17], whereas other studies report increased FA [18–20] or low sensitivity within the same region [21–24]. Changes in other DTI indices in PD in white matter structures of the brain associated with PD pathology have also been described previously, suggesting that white matter neurodegeneration (due to PD) may be detectable using DTI [25]. Basal ganglia group differences in DTI metrics have also been reported, some demonstrating decreased FA [13, 15], however others report no change [11]. Similarly, there are conflicting reports regarding the relationship between PD motor symptoms and DTI-derived parameters [11–13, 15, 26–28], suggesting that the DTI parameters may not be sensitive enough for characterization of PD CNS tissue changes alone.

Diffusion measurements using the DTI model relies on a Gaussian unrestricted diffusion model weighted predominantly by free and hindered diffusion. However, as the underlying microstructure within the grey matter is heterogeneous and unorganized, and diffusion uniformly restricted, the Gaussian model may have limited sensitivity in these regions. Tissue characterization of the underlying microstructure may thus benefit from a diffusion model accounting for heterogeneous compartmentalization at the sub-voxel level. Published studies using advanced modelling on PD data are few, however studies using the diffusion kurtosis imaging technique (DKI) [29], which quantifies the degree of heterogeneity of the tissue, have suggested improved MR-based diagnosis of the basal ganglia and substantia nigra of the grey matter [10, 19, 29–32].

An extended diffusion modelling technique named Restriction Spectrum Imaging (RSI) [33] uses high-angular DWI data obtained across a range of b-values to separate intracellular restricted diffusion from hindered diffusion across different length scales and tissue geometries. RSI has been shown to give valuable information on tissue cellularity, and for neurological diseases it may allow within-voxel characterization of neurite densities and organization [34–36]. The technique has, however, never been used to study PD pathology prior to this study. As brain-stem and cortical pathology may be sites affected early in Lewy Body disease, and MRI techniques with sufficient specificity and sensitivity for PD pathology at these sites have not been developed, the aim of this study was to evaluate both DTI and RSI measurements and their ability to detect potential PD in these regions. By isolating the restricted diffusion volume fraction from the hindered diffusion, the RSI model allows us to investigate restricted compartments separately, which may hold important information about the underlying tissue microenvironment not available using DTI.

## Materials and methods

### Study population

The study was approved by the Ethical Committee of the Medical Faculty of Masaryk University in Brno, Czech Republic, with approval number: 32/2014. All participants signed an informed consent form.

For the purpose of this study a total of 100 individuals were enrolled based on published research criteria [1], of them 46 PD patients (age: 63.1 ± 9.4, 14 females, details in Table 1) and 54 healthy controls (HC) (age: 67.2 ± 7.2, 37 female) (Details in Table 1). All were examined by both an experienced neurologist and an experienced neuropsychologist using a detailed neuropsychological cognitive test battery [37, 38]. Subjects with any diagnosed psychiatric disorder, dementia or subjects with alcohol/drug abuse were excluded from the study. All patients were longitudinally followed at the First Department of Neurology, Masaryk University and St Anne’s Hospital, Brno, Czech Republic.

**Table 1:** Demographics and clinical characteristics.

**PD**	
Number of subjects	46
Age at examination, years	63.1 ± 9.4, range 43–86
Females	14 (30.4%)
Years with symptoms	5.5217, range 1–20
UPDRS3	17.7826, range 5–40
**HC**	
Number of subjects	54
Age at examination, years	67.2 ± 7.2, range 47–81
Females	37 (68.5%)

Demographics and clinical characteristics of the study population with n = 100

### MRI

MRI was carried out on a 3T Siemens Prisma scanner with a 64-channel receiving head-neck coil. T1 images were acquired by Ultrafast Gradient Echo 3D (MPRAGE) sequence with TR 2300 ms, TE 2.36 ms, TI 900 ms Echo, voxel size 1x1x1 mm, FOV 256x256x240 mm, PAT 2 GRAPPA, flip angle 8°, Turbo factor 240. For DWI EPI based sequence we used TR 9300 ms, TE 97 ms, voxel size 2x2x2 mm, FOV 228x228x130 mm (matrix 114x114x65, with fat suppression), EPI factor 114, PAT 2 GRAPPA, Diffusion mode MDDW with 1 b = 0 image and 30 non-collinear diffusion gradient directions for each diffusion weightings of b = 500, 1000, 2000, 4000 s/mm^2^. In addition, a single SE-EPI volume was acquired at b = 0 and with opposite phase direction to allow for EPI-distortion correction [39].

### Image processing and statistical analysis

#### Image processing and model estimation

All image processing, except the RSI analysis, was carried out using the FMRIB Software Library v5.0 (FSL) [40] and Matlab R2015b (MathWorks, Natick, Massachusetts).

Brain extraction, EPI-distortion, eddy-current and movement corrections were done using the *bet*, *topup* and *eddy* toolboxes in FSL. The quality of all corrected output data was evaluated by visual inspection. A standard tensor model was fit to the DW-data using *dtifit* from the FSL toolbox [41]. Here, the DTI model was modelled using b = 0 and b = 1000 s/mm^2^. The descriptive diffusion parameters FA, MD, Axial (AD) and Radial Diffusivity (RD) were derived. RSI models the high-angular DW data based on the parameterization of the fiber orientation density function (FOD) using 4^th^ order spherical harmonics, combined with an axially-symmetric Gaussian model [33]. The RSI spectrum was fit to the DW data using least-squares estimation with Tikhonov regularization. The regularization constant was determined based on careful evaluating the root mean square deviation of the modelled signal attenuation compared to the measured signal attenuation over a range of constants. The RSI spectrum was defined as a combination of anisotropic, restricted and free/hindered diffusion compartments as detailed by White et al [33]. Here, the longitudinal diffusivity (D_L_) is fixed to 3.4·10^−3^ mm^2^/s and the transverse diffusivity (D_T_) was varied from 0 mm^2^/s to D_L_ in 10 equally spaced steps. In addition, two extra terms were added to the model; spherically restricted water diffusion defined as D_T_ = 0 and D_L_ = 0, and free water diffusion defined as D_T_ = D_L_ = 3.4·10^−3^ mm^2^/s. For the purpose of this study we focused the analysis on the water signal fraction from the spherically restricted diffusion compartment (cellularity index; CI) and the water signal fraction of anisotropic restricted diffusion by summing the scales of D_T_/D_L_ = 0 and D_T_/D_L_ = 0.225, assumed to reflect relative density of neuronal processes (defined here as "nurite density"; ND) [33]. RSI modelling was performed in Matlab.

#### Co-registration

To prepare for voxel-wise and ROI based analysis, all structural and DW parametric maps were co-registered into a common space using the FSL software library. First, all T1 weighted (T1w) images were grey matter (GM) segmented and registered to the MNI 152 standard space using non-linear registration [42]. A carefully chosen selection of gender and age matched structural images from both groups of patients and controls were averaged to create a study-specific GM template [43]. All individual T1w images were subsequently registered to the GM template. All the b = 0 images from the DWI set were registered to the corresponding T1w image using affine 12 degrees of freedom registration, and further registered to the common average GM template using the same T1w-to-GM template transformation. All diffusion parametric maps derived from the DWI set (FA, MD, AD, RD, CI and ND) underwent the same transformations derived for the b = 0 images. The co-registered diffusion parametric maps were lastly smoothed using Gaussian kernel of FWHM = 3 mm.

#### Defining regions of interest

**Fig 1:**
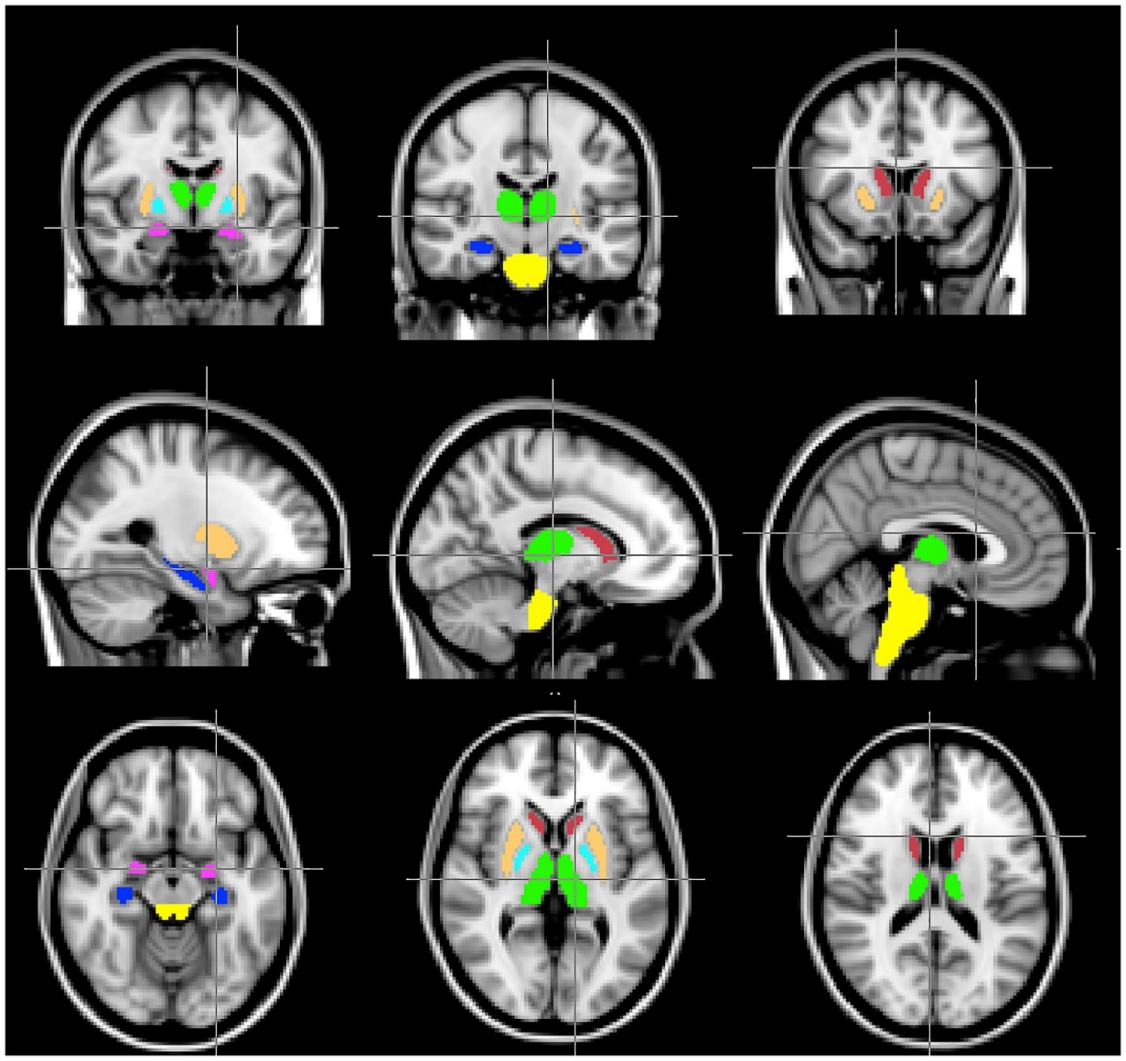
ROIs evaluated in the study. Thalamus (green), hippocampus (blue), brainstem (yellow), amygdala (magenta), palladium (beige), caudate (dark red) and putamen (cyan).

The main focus in this study was assessing group differences in the subcortex and brainstem. For this purpose, voxel-wise analysis was performed within the brainstem (2743 voxels), thalamus ((left; right) of 758 and 752 voxels respectively), caudate (left; right 193; 198 voxels), palladium (left; right 101; 111 voxels), putamen (left; right 443; 431 voxels) and amygdala (left; right 95; 114 voxels). We also examined the hippocampus (left; right, 214; 183 voxels) as cognitive impairment is frequent in PD and we have previously demonstrated smaller hippocampi in PD as compared to controls [44]. All ROIs analysed in the study were defined and implemented by the FSL Harvard-Oxford subcortical structural atlas and are displayed in Fig 1. The ROIs were probabilistically defined and thresholded at 80% (i.e > 80% likelihood of a voxel analysed being within the specific region).

#### Statistical analysis

Voxel-wise group differences for each diffusion parameter were analyzed within each ROI by applying general linear modelling (GLM) using *randomise*, a FSL statistical tool. *Randomise* allows for permutation-based non-parametric testing using cluster-based thresholding to correct for multiple comparisons across space. *Randomise* was applied to the co-registered and smoothed parametric maps using 5000 permutations and a cluster-based correction threshold of t > 2.3. Age was regressed out for all analyses. Due to a small sample size there was a slight mismatch in the distribution of genders between the control group and the PD group (Table 1), hence gender was *not* regressed out in this analysis. We recognize this as a limitation. To account for the difference in separability between the different parameters, we estimated the degree of separability (AUC; Area Under Curve) between groups in significant regions using Receiver Operator Characteristics (ROC).

## Results

We found that that both DTI-derived and RSI-derived metrics are sensitive to group effects with significant clusters (p < 0.05) in the thalamus, the hippocampus and the amygdala manifested as increased CI, MD, RD and AD and decreased ND in the PD group. CI was also significantly increased in the brainstem of the PD group. The AUC was largest using CI (0.66–0.69) with a larger group difference (~ 8%) compared to the remaining metrics (~ 0.55, ~ 3%), suggesting that CI may be more sensitive to pathological changes in the PD group.

**Fig 2:**
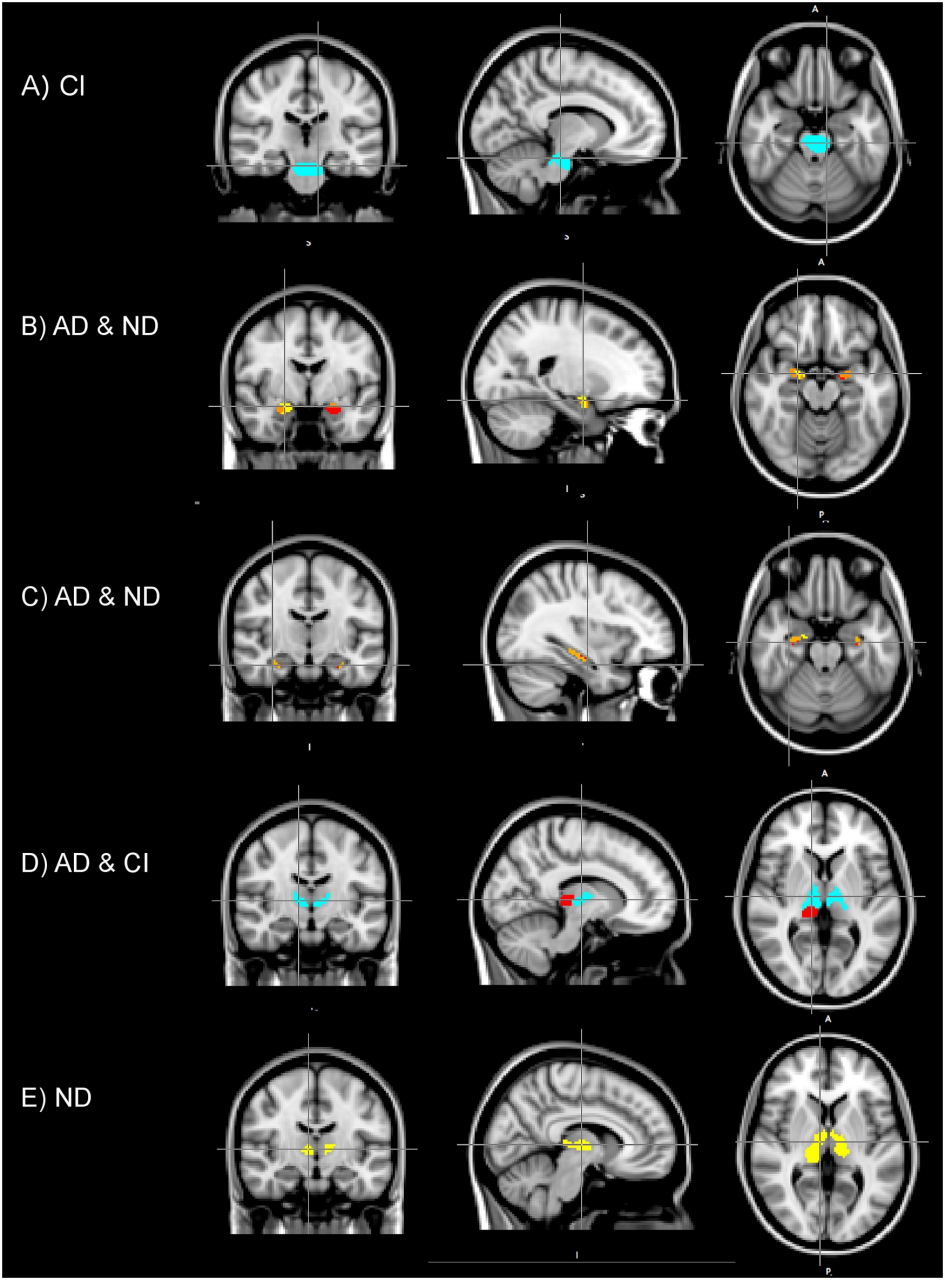
Results of the voxel-wise group analysis. Brainstem (A), the amygdala (B), the hippocampus (C) and the thalamus (D, E). Images display clusters of voxels where group differences are significant (p<0.05). Here, CI and AD were increased, and ND was decreased in the patient group in the respected clusters. A) The cluster in the brainstem where CI were increased. C) AD (red) compared with ND (yellow), overlapping regions of significance (orange). D) AD (red) compared with ND (yellow) and joint (orange) C) AD (red) and CI (magenta), no overlapping voxels. D) ND (yellow).

The significant results of the voxel-wise analysis and corresponding AUCs are summarized in Table 2. Here, mean values of each group and relative group difference (PD/HC), absolute mean values of each group, AUC and relative number of significant voxels (p < 0.05) compared to the full ROI size (100 * # significant voxels/ # voxels in the ROI) within each cluster are displayed. Only ROIs with significant group difference are shown. Clusters with significant group differences after accounting for multiple comparisons (p<0.001) are denoted with an asterisk (*). Fig 2 displays clusters of significant group difference at p < 0.05, and t > 2.3. Only CI was sensitive to group differences in the brainstem, significant clusters are displayed in Fig 2A. Fig 2B–2E show significant clusters using ND, AD and CI for the sub cortical regions; thalamus, hippocampus and amygdala. Significant regions using the remaining metrics were similar in size to the regions depicted here (see statistical summary in Table 2).

**Table 2:** Differences between patients (PD) and healthy controls (HC).

	AD	MD	RD	ND	CI
**Brainstem**					
PD/HC	-	-	-	-	1.075* (PD: *0*.*17 ± 0*.*03)* *(HC*: *0*.*16 ± 0*.*03)*
AUC					0.69
%					21.60%
**Right thalamus**					
PD/HC	1.018 *(PD*: *1*.*35 ± 0*.*30)* *(HC*: *1*.*33 ± 0*.*30)*	1.020 *(PD*: *1*.*18 ± 0*.*30)* *(HC*: *1*.*16 ± 0*.*30)*	1.022 *(PD*: *1*.*17 ± 0*.*35)* *(HC*: *1*.*14 ± 0*.*35)*	0.97* (PD: *0*.*62 ± 0*.*13)* *(HC*: *0*.*63 ± 0*.*13)*	1.071 *(PD*: *0*.*12 ± 0*.*03* *(HC*: *0*.*11 ± 0*.*03)*
AUC	0.55	0.55	0.55	0.59	0.67
%	19.70%	16.20%	15.80%	41.10%	31.80%
**Left thalamus**					
PD/HC	-	-	-	0.978* (PD: *0*.*69 ± 0*.*10)* *(HC*: *0*.*70 ± 0*.*10)*	1.071 *(PD*: *0*.*12 ± 0*.*03)* *(HC*:*0*.*11 ± 0*.*03)*
AUC				0.59	0.67
%				30.90%	27.40%
**Right hippocampus**					
PD/HC	1.035 *(PD*: *1*.*35 ± 0*.*20)* *(HC*: *1*.*31 ± 0*.*20)*	1.037 *(PD*: *1*.*16 ± 0*.*20)* *(HC*: *1*.*11 ± 0*.*20)*	1.040 *(PD*: *1*.*05 ± 0*.*40)* *(HC*: *1*.*01 ± 0*.*30)*	0.967* (PD: *0*.*58 ± 0*.*11)* *(HC*: *0*.*60 ± 0*.*10)*	-
AUC	0.55	0.54	0.54	0.55	
%	45.54%	50.82%	54.10%	48.63%	
**Left hippocampus**					
PD/HC	1.039 *(PD*: *1*.*33 ± 0*.*30)* *(HC*: *1*.*28 ± 0*.*20)*	1.041 *(PD*: *1*.*14 ± 0*.*20)* *(HC*: *1*.*09 ± 0*.*20)*	1.045 *(PD*: *1*.*05 ± 0*.*50)* *(HC*: *1*.*00 ± 0*.*30)*	0.964 *(PD*: *0*.*57 ± 0*.*11)* *(HC*: *0*.*59 ± 0*.*10)*	-
AUC	0.54	0.54	0.53	0.55	
%	21.03%	19.16%	17.29%	15.89%	
**Right amygdala**					
PD/HC	1.022* (PD: *1*.*23 ± 0*.*20)* *(HC*: *1*.*21± 0*.*20)*	1.022* (PD:*1*.*03 ±0*.*20)* *(HC*:*1*.*01± 0*.*20)*	1.022* (PD:*0*.*94 ± 0*.*40)* *(HC*: *0*.*92± 0*.*40)*	0.973* (PD: *0*.*63 ± 0*.*14)* *(HC*: *0*.*64 ± 0*.*13)*	1.084 *(PD*: *0*.*05 ± 0*.*001)* *(HC*: *0*.*05 ± 0*.*01)*
AUC	0.55	0.55	0.55	0.57	0.66
%	47,37%	52,63%	50%	88,60%	17.54%
**Left amygdala**					
PD/HC	1.025* (PD: *1*.*28 ± 0*.*20)* *(HC*: *1*.*25 ± 0*.*20)*	1.029* (PD:*1*.*04 ± 0*.*20)* *(HC*: *1*.*01± 0*.*20)*	1.034* *(PD*: *0*.*85 ± 0*.*40)* *(HC*: *0*.*92 ± 0*.*40)*	0.975* (PD:*0*.*60 ± 0*.*09)* *(HC*:*0*.*61 ± 0*.*09)*	-
AUC	0.58	0.59	0.60	0.57	
%	82.11%	89.47%	86.32%	37.89%	

The results of the voxel wise analysis, showing relative group difference (PC/HC), along with mean absolute value for each group, measured separability (AUC) and the percentage of significant voxels (p<0.05) compared to the full ROI size for each ROI and diffusion metric.

‘-‘ denotes when there is no significant group difference.

‘*’ denotes parameters that were significant when accounting for multiple comparisons (p<0.001). The DTI-metrics are in the unit 10^-3^mm^2^/s while the RSI-metrics are unitless.

## Discussion

In this study, we investigated two diffusion-based MRI techniques and their sensitivity to microstructural changes within brain regions associated with motor function in PD. Group differences between a PD patient group and an age-matched HC group have been analyzed using both standard DTI-derived metrics (assuming Gaussian diffusion), and diffusion metrics describing the restricted non-Gaussian diffusion fraction derived from the RSI model.

In summary, we found that both diffusion-based models were sensitive to group specific differences in subcortical brain regions known to be prone to pathology in PD. Our results further indicate that CI and ND may improve detection of between group differences, and provide additional information when combined with the remaining diffusivity measurements.

DTI-derived parameters are estimated based on a Gaussian diffusion model, and is commonly associated with extracellular, hindered water diffusion. Increased RD and AD (and MD) is typically considered an effect of atrophy, demyelination or loss of structural organization due to an increased extracellular space [45–49]. Our findings are consistent with previous reports of increased diffusivity in regions known to be affected by PD pathology. In contrast, the RSI-derived parameters CI and ND are assumed to reflect the signal fraction from isotropic and anisotropic restricted diffusion pools associated with intracellular space, hence changes in these metrics may be related to microstructural and axonal changes. As ND reflect the fraction of restricted anisotropic diffusion, assumed related to the neurite density [33, 50], decreased ND might implicate microstructural damage of- or related to the neurons. Increased CI, interpreted as increased isotropic restriction, may be related to glia infiltration or activation, as previously shown in the PD brainstem [3, 51]. Although the true biological interpretation of either is currently vague, the findings fit well with the current understanding of ND and CI biological underpinnings, and the understanding of PD pathophysiology. We argue that changes measured using the two models reflect different aspects of neurodegeneration and that the RSI model hold relevant information about the underlying tissue microenvironment not available using DTI. The RSI technique (or other non-Gaussian models) may be considered a supplementary model, contributing a fuller picture of the underlying pathology.

Neurodegeneration of thalamic regions is known to occur in association with loss of dopaminergic function in the basal ganglia in PD and there has been substantial evidence of PD pathology within the thalamus [52]. Previous studies have reported increased MD and decreased FA as a result of PD pathology within the thalamus [13, 28]. In this study, we found no evidence that FA significantly differed between groups, however, diffusivity (MD, RD and AD) was increased in the thalamus of the PD group. Furthermore, we found that CI and ND were changed in the PD group, suggesting loss of microstructural and neuronal integrity in the thalamus. Similar results were found in the amygdala and hippocampus as well, suggesting that all these regions experience some extent of neurodegeneration at the microstructural level in addition to demyelination and loss of structural organization.

As mentioned in the introduction, despite reports suggesting that FA may be a sensitive biomarker for tracking pathological changes within e.g. the substantia nigra [10–17], other reports have contradicted these findings [18–20]. As the subcortical regions are structurally heterogeneous and unorganized, however, neurodegeneration would likely occur correspondingly. As a consequence, MD, AD and RD should be more sensitive to degeneration and damage of these regions compared to FA, which is also in accordance with our analysis. Regardless, as our results are limited by a lower image resolution of the brainstem compared to other previous studies of the substantia nigra, our study cannot confirm nor contradict previous findings. Using an adapted high-resolution DTI protocol may improve sensitivity for detecting pathology particularly within the brainstem.

No significant group difference was found within the pallidum or striatum using either of the techniques. As the organization of neurons in the basal ganglia is known to be atypical and less myelinated compared to white matter regions, the DTI-derived metrics may simply be insensitive to changes in these regions. Similarly, RSI measures did not reveal group differences in these regions either, which may be due to variations between basal ganglia neuropathology and neuropathology at other PD predilection sites, e. g. in terms of glia activation, or aspects of neurodegeneration.

Limitations of the present study include potential influence of anti-Parkinson medication and neurostructural differences related to the sex, and years with PD diagnosis which were not used as regressors due to the small sample size. Also, longitudinal clinical and imaging data is not available at present but is planned for a future work. To avoid overfitting, ROI volume size was not accounted for in the primary GLM analysis as a multivariate analysis showed that diffusion metrics were more strongly linked to group differences compared to the ROI volume, and average volume size within a group did not significantly differ between groups. In parallel, however, we repeated the GLM analysis including volume as variable. These results yielded similar results as the results stated above, but significant clusters were slightly smaller in size as a result of volume effects. Although the presented approach has potential limitation, we argue that it does not interfere with our hypothesis that RSI provides supplementary information about tissue microstructure and pathology as both RSI and DTI are introduced to the same bias, whereas differences between the two remain the same.

Parkinson pathology is generally varied, both during the disease course and between affected sites in an individual patient. In the absence of major atrophy, our study suggest that diffusion techniques may reveal microstructural pathology and protocols for MRI diffusion imaging of PD may be adapted to more sensitive detection of pathology at different CNS sites.

## Conclusions

In conclusion according to our analyses, both DTI and RSI are sensitive to PD pathology. Changes measured using the two models reflect different aspects of neurodegeneration and should be considered complimentary techniques. Adapting protocols for more sensitive detection of PD pathology may improve detection and understanding of pathology.
